# The Feasibility of Differentiating Lewy Body Dementia and Alzheimer’s Disease by Deep Learning Using ECD SPECT Images

**DOI:** 10.3390/diagnostics11112091

**Published:** 2021-11-12

**Authors:** Yu-Ching Ni, Fan-Pin Tseng, Ming-Chyi Pai, Ing-Tsung Hsiao, Kun-Ju Lin, Zhi-Kun Lin, Chia-Yu Lin, Pai-Yi Chiu, Guang-Uei Hung, Chiung-Chih Chang, Ya-Ting Chang, Keh-Shih Chuang, Alzheimer’s Disease Neuroimaging Initiative

**Affiliations:** 1 Health Physics Division, Institute of Nuclear Energy Research, Atomic Energy Council, Taoyuan 325, Taiwan; ecyor@iner.gov.tw (F.-P.T.); zklin@iner.gov.tw (Z.-K.L.); chiayulin@iner.gov.tw (C.-Y.L.); 2 Department of Biomedical Engineering and Environmental Sciences, National Tsing-Hua University, Hsinchu 300, Taiwan; kschuang@mx.nthu.edu.tw; 3 Division of Behavioral Neurology, Department of Neurology, National Cheng Kung University Hospital, College of Medicine and Institute of Gerontology, National Cheng Kung University, Tainan 701, Taiwan; 4 Alzheimer’s Disease Research Center, National Cheng Kung University Hospital, Tainan 704, Taiwan; 5 Department of Medical Imaging and Radiological Sciences & Healthy Aging Center, Chang Gung University, Taoyuan 333, Taiwan; ihsiao@mail.cgu.edu.tw (I.-T.H.); kunjulin@gmail.com (K.-J.L.); 6 Department of Nuclear Medicine and Molecular Imaging Center, Linkou Chang Gung Memorial Hospital, Taoyuan 333, Taiwan; 7 Department of Neurology, Show Chwan Memorial Hospital, Changhua 500, Taiwan; paiyibox@gmail.com; 8 Department of Nuclear Medicine, Chang Bing Show Chwan Memorial Hospital, Changhua 505, Taiwan; 106143@gmail.com; 9 Department of Neurology, Kaohsiung Chang Gung Memorial Hospital, Kaohsiung 833, Taiwan; neur099@cgmh.org.tw; 10 Department of Neurology, Institute of Translational Research in Biomedicine, Kaohsiung Chang Gung Memorial Hospital, Chang Gung University College of Medicine, Kaohsiung 833, Taiwan; emily060634@gmail.com

**Keywords:** ECD SPECT images, Lewy body dementia, Alzheimer’s disease, transfer learning

## Abstract

The correct differential diagnosis of dementia has an important impact on patient treatment and follow-up care strategies. Tc-99m-ECD SPECT imaging, which is low cost and accessible in general clinics, is used to identify the two common types of dementia, Alzheimer’s disease (AD) and Lewy body dementia (LBD). Two-stage transfer learning technology and reducing model complexity based on the ResNet-50 model were performed using the ImageNet data set and ADNI database. To improve training accuracy, the three-dimensional image was reorganized into three sets of two-dimensional images for data augmentation and ensemble learning, then the performance of various deep learning models for Tc-99m-ECD SPECT images to distinguish AD/normal cognition (NC), LBD/NC, and AD/LBD were investigated. In the AD/NC, LBD/NC, and AD/LBD tasks, the AUC values were around 0.94, 0.95, and 0.74, regardless of training models, with an accuracy of 90%, 87%, and 71%, and F1 scores of 89%, 86%, and 76% in the best cases. The use of transfer learning and a modified model resulted in better prediction results, increasing the accuracy by 32% for AD/NC. The proposed method is practical and could rapidly utilize a deep learning model to automatically extract image features based on a small number of SPECT brain perfusion images in general clinics to objectively distinguish AD and LBD.

## 1. Introduction

Alzheimer’s disease (AD) is the most common type of dementia in neurodegenerative diseases of the brain, accounting for more than 60% of all dementia cases [1], followed by Lewy body dementia (LBD), which accounts for about 10–30% of all cases of dementia [1,2]. Although there are consensus criteria for the clinical diagnosis of both diseases [3,4], in some cases, due to the overlap of clinical and pathological features, it may be difficult to distinguish between LBD and AD patients. Early diagnoses of LBD and AD are important from prognostic and therapeutic perspectives, and distinguishing them is clinically vital [5]. Functional imaging methods, such as F-18-FDG (fluorodeoxyglucose, FDG) PET and cerebral perfusion SPECT are considered useful for clarifying the diagnosis of dementia. Although increasingly more specific ligands are available (e.g., amyloid), the mainstay of functional brain imaging for the differential diagnosis of dementia remains F-18-FDG PET and cerebral perfusion SPECT for the foreseeable future [6]. The studies of SPECT brain perfusion and PET metabolism are usually consistent in abnormal areas [7]. However, F-18-FDG PET, which is typically used in the West for brain glucose metabolism examination, is currently not covered by the National Health Insurance in Taiwan [8]. Therefore, most nuclear medicine departments in Taiwan use cerebral perfusion SPECT and the Tc-99m-ECD (ethyl cysteinate dimer, ECD) tracer. Although SPECT has a longer imaging time and poorer image resolution than PET, it is low cost and the tracer is easily accessible; hence, it is widely used in domestic clinical practice. Therefore, this study focused on how to use Tc-99m-ECD SPECT images to differentiate between LBD and AD.

Previous studies have shown that the abnormal areas of F-18-FDG PET and cerebral perfusion SPECT images of AD patients are usually bilateral temporoparietal areas, posterior cingulate, and medial temporal areas, with sensory-motor cortices, including the cerebellum, largely spared [9,10]. However, the abnormal areas of LBD and AD often overlap. O’Brien et al. compared F-18-FDG PET and cerebral perfusion SPECT imaging [tracer: Tc-99m-HMPAO (hexamethyl propylene amine oxime, HMPAO)] in the differential diagnosis of AD and LBD, showing that the area under the ROC curve (AUC) of F-18-FDG PET and cerebral perfusion SPECT were 0.8 and 0.58 [6]. In addition, hypoperfusion in the posterior cingulate cortex (PCC) was observed in AD, whereas the PCC is relatively preserved in LBD. The phenomenon of sparing the PCC relative to the precuneus plus cuneus, termed the cingulate island sign (CIS) [11], has recently garnered attention because it reflects concomitant AD pathology that affects the clinical symptoms of LBD [12,13]. Imabayashi et al. developed a discrimination method using optimized VOI (focus on the occipital lobe and cingulate cortex) on cerebral perfusion SPECT images (tracer: Tc-99m-ECD), with 92.3% sensitivity and 76.9% specificity [14]. Shimizu et al. studied cerebral perfusion SPECT images [tracer: I-123-IMP (N-isopropyl-p-[I-123] iodoamphetamine, I-123-IMP)] analyzed by three-dimensional stereotactic surface projections (3D-SSP), cerebral perfusion in the medial occipital lobe, and distinguished LBD from AD with 85% sensitivity and specificity [5]. Iizuka et al. used the 3D-SSP analysis of I-123-IMP SPECT images for convolutional neural network (CNN) model training, with an 89% accuracy of distinguishing LBD and AD [15]. The literature shows that the use of cerebral perfusion SPECT imaging (tracers including I-123-IMP, Tc-99m-ECD, Tc-99m-HMPAO) can distinguish AD, NC, and LBD; however, as abnormal areas of AD and LBD images often overlap, it is difficult to distinguish between the two. Hence, additional image processing and analysis are required to improve the discrimination, such as calculated specific VOI values and a Z-score surface map of the 3D-SSP images, etc.

In recent years, due to the advanced digitalization of medical data, novel technologies, studies applying artificial intelligence (AI), radiomics technology in medical imaging, and the identification of noninvasive disease features have increased significantly [16]. Current data featuring learning methods can automatically discover features in the original data and generate insights [17]. For example, the deep learning algorithm transforms the original data into more detailed features through the nonlinear function composed of a hierarchical structure, thereby identifying new patterns [18]. Multidisciplinary clinical neuroscience has begun to be influenced by deep learning and is moving toward the development of new diagnostic and prognostic tools. Indeed, deep learning technology is particularly promising in neuroscience because clinical diagnosis usually relies on subtle symptoms and complex neuroimaging methods [19].

Deep learning technology can automatically extract features from the original data, but a large amount of data needs to be prepared for deep learning model training [20]. Compared with thousands or tens of thousands of X-ray chest imaging databases, the number of nuclear medicine brain images is much smaller. Although nuclear medical imaging is a highly sensitive functional image modality, the tracer used can directly reflect the distribution of biomarkers in the brain and effectively detect neurological diseases, but the small number of nuclear medical images limits the research using deep learning technology in this field. In the field of neuroscience, many researchers have applied deep learning on magnetic resonance imaging to detect AD [21], and some have applied functional magnetic resonance imaging, magnetoencephalography, and electroencephalography signals to detect AD [22]. Only a few have applied deep learning on nuclear medicine imaging to distinguish AD [23,24,25], with most using F-18-FDG PET images from the ADNI public database. The authors’ previous study using two-stage transfer learning technology via F-18-FDG PET images of the public database ADNI successfully distinguished AD and NC from the Tc-99m-ECD SPECT images, overcoming the issue of a small amount of data. The study indicated that the model trained on PET FDG metabolic imaging for the same disease could be transferred to a small sample of SPECT cerebral perfusion images [26]. However, apart from AD, there is almost no relevant research on nuclear medicine images applied to deep learning technology to distinguish other types of dementia.

In this study, we aimed to evaluate whether the deep learning models could be trained to distinguished AD from LBD using a real clinical data set, a small amount of Tc-99m-ECD SPECT images. Our previous study proved that in AD/NC classification tasks, feature extraction from a relatively large number of F-18FDG PET image data sets can be transferred to a relatively small number of Tc-99m-ECD SPECT image data sets to overcome data size. We further investigated whether such models can be applied to an independent data set with different disease domains and LBD patients to differentiate AD and LBD.

## 2. Materials and Methods

### 2.1. Subjects

Tc-99m-ECD SPECT images (total 308 subjects: 134 NC, 113 AD, 61 LBD) from the Taiwanese Nuclear Medicine Brain Image database were collected and built by the Institute of Nuclear Energy Research. All participants were evaluated by neurologists and clinical psychologists, and their education level was elementary school or above. People with normal cognitive function were assessed to rule out physical conditions that cannot be corrected and may cause dementia or delirium, such as poor vision, abnormal hearing, hypothyroidism, anemia, pneumonia, fever, dehydration, signs of abnormal liver function, abnormal renal function, signs of heart failure (NY class < 3), etc. Those with obvious head trauma, neurological diseases related to dysfunction of the extrapyramidal system or autonomic nervous system, such as hydrocephalus, Parkinson’s disease, cortical basal ganglia degeneration, and progressive supranuclear palsy, Vitamin B12 or folic acid deficiency caused by subacute combined degeneration, multiple system degeneration, and cerebrovascular diseases that may cause various local neurological symptoms were excluded. The systolic pressure of those with hypertension needed to be controlled below 160 mmHg, and the HbA1c of those with diabetes mellitus below 9.0. Those on medications that may cause cognitive dysfunction, such as anticholinergic drugs, hypnotics, or antipsychotics, were excluded. The critical mental illness scale (CHQ-12) score should be < 3, and all participants completed the clinical dementia rating (CDR) scale to determine the severity. Participants with clinically suspected AD or LBD received a complete medical history inquiry (including important system and brain disease history and CDR), cognitive function (such as Mini-Mental State Examination (MMSE) scores), and related examinations. Those who met the criteria further underwent Tc-99m-ECD SPECT imaging, and the images were interpreted by nuclear medicine experts. The demographic characteristics and clinical characteristics are shown in Table 1. The Institutional Review Board (IRB) of National Cheng Kung University Hospital approved this study (serial number: NCKUH IRB B-BR-107-030).

The F-18-FDG PET images of AD and NC (total 1333 subjects: 666 NC, 667 AD) used for pretraining in this study were obtained from ADNI, a public database, and the demographic and clinical characteristics of the data are shown in Table 2.

### 2.2. Image Acquisition and Processing

F-18-FDG PET images were downloaded from the ADNI database (http://adni.loni.usc.edu). The ADNI was launched in 2003 as a public–private partnership, led by Principal Investigator Michael W. Weiner, MD. The primary goal of ADNI has been to test whether serial magnetic resonance imaging (MRI), PET, other biological markers, and clinical and neuropsychological assessment can be combined to measure the progression of mild cognitive impairment (MCI) and AD. These images were preprocessed to achieve database consistency. All F-18-FDG PET images were spatially normalized into the MNI space with an image size of 91 × 109 × 91 using the registration method and FDG template in SPM8 software (University College of London, London, UK). After spatial normalization, these images were further cropped, padded, and the image slices above the skull and below the cerebellum were removed to retain most of the brain parenchymal area, with a final image size of 95 × 95 × 48.

Tc-99m-ECD SPECT images were acquired from four medical institutions and obtained by E-CAM, Symbia T16, and Symbia T2 SPECT equipment (Siemens Medical Solutions, Malvern, PA, USA) with LEHR (low energy high resolution) and fan beam collimators. Fifteen minutes after intravenous injection of 925 MBq Tc-99m-ECD, SPECT images were acquired for 30 to 40 min, and the image matrix size was 128 × 128. The images were reconstructed by filtered back projection (FBP) with a Metz filter and ordered subsets expectation and maximization (OSEM) method using Chang’s attenuation correction (attenuation coefficient is 0.1 cm^−1^). The original image was processed for spatial normalization using the registration method and SPECT perfusion template in SPM8 software. These SPECT images were intensity normalized using the Z-score method. The image values were scaled to a distribution with an average value of zero and a standard deviation of one. The image was resampled to 95 × 95 × 68 with the voxel size 2 × 2 × 2 mm^3^, and the image slices above the cranium and below the cerebellum were removed to retain most of the parenchymal area, giving a final image dimension of 95 × 95 × 48.

**Figure 1:**
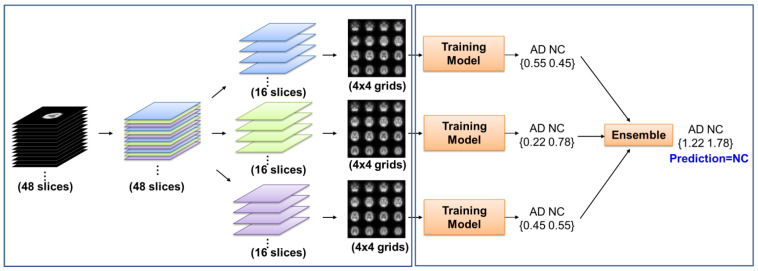
Schematic diagram of the 3D image divided into three 2D images for training.

To effectively use the computing resources during training and adopt the pretraining model used in the image vision field, 3D medical images needed to be reduced to 2D images. To retain the information of the whole brain slices, the brain parenchymal area with an image dimension of 95 × 95 × 48 was equally divided into 16 sections, with one image selected for each section, then 4 × 4 slices were reassembled to a 2D image. A 3D image (F-18-FDG PET or Tc-99m-ECD SPECT) was divided into three 2D images with a 380 × 380 matrix size. Sixteen slices were sorted in order from the caudal of the brain to the cranial as shown in the left part of Figure 1.

### 2.3. Pretrained and Training Model

This research is based on a simple computing device environment and a limited amount of SPECT data to investigate how to overcome the above disadvantages and successfully train via deep learning technology to achieve the goal of nuclear medicine imaging disease classification. The differentiation of dementia using Tc-99m-ECD SPECT images included distinguishing AD and NC, LBD and NC, and AD and LBD. Our training strategy was to use ResNet-50, a commonly used model in the field of image visual classification, to perform Tc-99m-ECD SPECT image classification tasks for the assembled 2D images. Through the above training, the model could learn low-level image features. Then F-18-FDG PET images from ADNI were used for the pretrained. With such transfer learning, the model could learn not only low-level image features but also the features of nuclear medicine images. Finally, we modified the model by reducing the complexity to improve the training performance.

**Figure 2:**
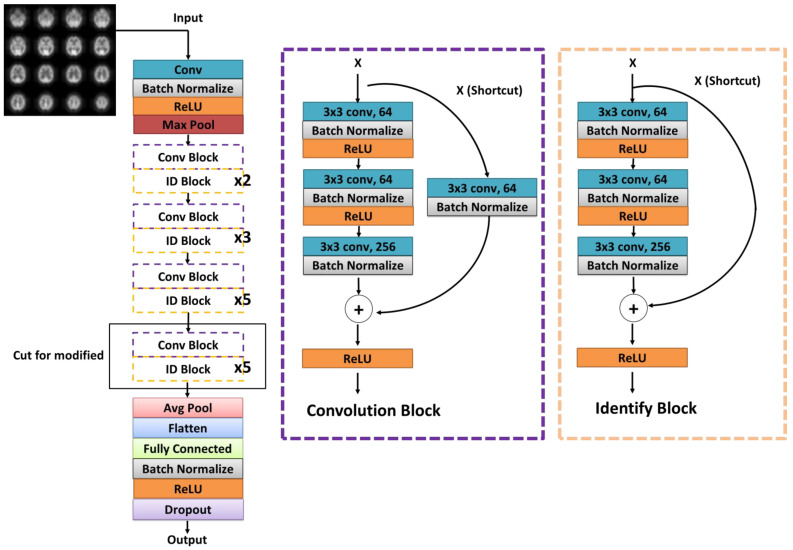
The convolutional neural network architecture, ResNet-50, used in this study.

In the study, the effects of the three training methods were compared: (*a*) *Original ResNet-50*, which loaded the ResNet-50 weights trained on the ImageNet data set. In Figure 2, an average pooling was connected to the top layer of the ResNet-50 model, then the fully connected layer (FC) with a length of 128, the batch normalization (BN), and the dropout layer were added. The dropout layer was set to 0.5 as a form of regulation to avoid neural network coadaptation by randomly removing nodes for a more robust model. (*b*) *ResNet-50 with ADNI Pretrained*, which was first loaded with the ResNet-50 weights trained on ImageNet, then retrained by the F-18-FDG PET image data set, and finally retrained by the Tc-99m-ECD SPECT image data set with the weights of the aforementioned learning as the initial value. (*c*) *ResNet-50 with ADNI Pretrained* + *Modified*, which was the same as (b), but when transferring learning to the Tc-99m-ECD SPECT image, the model was modified to delete the high-level features corresponding to the fifth block of ResNet-50. The architecture of the training model is shown in Figure 2.

All training methods loaded the preprocessed image with a dimension of 380 × 380 on the computing machine of the Linux operating system (system version Ubuntu 18.04). In the container environment created by virtual technology, the resources were allocated with four cores of Intel Xeon 6230 2.1 GHz processor (Intel, Santa Clara, CA, USA), 48 GB of DDR4 memory, and an NVIDIA 2080Ti computing card (Nvidia Corporation, Santa Clara, CA, USA). The development environments were all executed under Python 3 using Keras 2.2.5 to build neural networks and import pretrained models, and the backend runs as TensorFlow 1.15.2 (Google, Mountain View, CA, USA).

We randomly selected 20% of the data from a classification task as an independent test set and the remaining 80% for training. The data comprised the ADNI pretrained database (F-18-FDG PET images), as well as Tc-99m-ECD SPECT images for AD/NC, LBD/NC, AD/LBD classification tasks, all of which were used in the same training/testing proportion (Table 3).

In the training process, data augmentation was used to increase the amount of training data and the tolerance of the training model to the data, preventing the neural network from memorizing training data to overcome the training problem of overfitting. The range of random width and height shift of data augmentation was 0–0.02% and the range of zooming was 1–1.03% for the Tc-99m-ECD SPECT images. The range of random width and height shift of data augmentation was 0–0.03% and the range of zooming was 1–1.03% for the F-18-FDG PET images.

The loss function used categorical cross entropy, and the optimization algorithm used adaptive moment estimation (Adam) [27], the learning rate was set to 0.0000005, and the batch size was set to 8 for model training. The early-stopping mechanism was used to judge the stop and choose a suitable epoch. The trained model was validated using 20% of the Tc-99m-ECD SPECT images and its performance was evaluated by accuracy to decide when to stop. For F-18-FDG PET images, all hyperparameters settings were almost the same as above, except for the learning rate was set to 0.000001.

Each 3D image was divided into three 2D images. The respective predicted probabilities of three 2D images from the same subject were summed for ensemble learning as shown in the right part of Figure 1.

### 2.4. Features Visualization

The nonlinear dimensionality reduction algorithm t-distributed stochastic neighbor embedding (t-SNE) [28] is suitable for dimension reduction of high-dimensional data to two dimensions for visualization. In this study, image features extracted from each image (including NC, AD, and LBD) through the deep learning model were dimension-reduced to two dimensions by t-SNE using package scikit-learn [29], allowing visual observation of the scattered location of each image to qualitative assess the similarity between the data.

### 2.5. Model Testing and Evaluation

The accuracy of the model was evaluated by receiver operating characteristic (ROC) curves and the AUC. The ROC curve was plotted with 95% confidence intervals (CI) calculated using MATLAB (MATLAB R2020a, MathWorks, Natick, MA, USA) with 1000 iterations of bootstrapping. In addition, statistical analysis was performed on the classification prediction results, including the calculation of the sensitivity, specificity, precision, accuracy, and F1 score. When calculating the above performance indicators of AD/NC, LBD/NC, and AD/LBD classification tasks, the category before the slash was defined as the positive class.

In addition, the author also listed all the research results of deep learning on cerebral perfusion SPECT images to distinguish AD/NC, LBD/NC, and AD/LBD. Although the training conditions of these studies were very different from ours (such as the tracer they used and data type after calculation), they were provided for reference.

## 3. Results

### 3.1. Features Visualization

**Figure 3:**
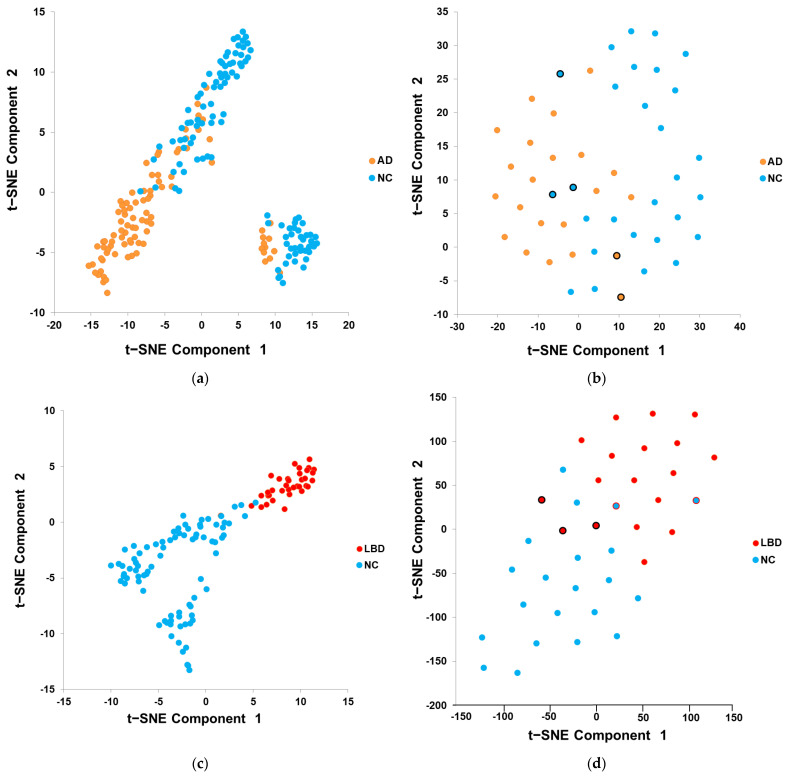

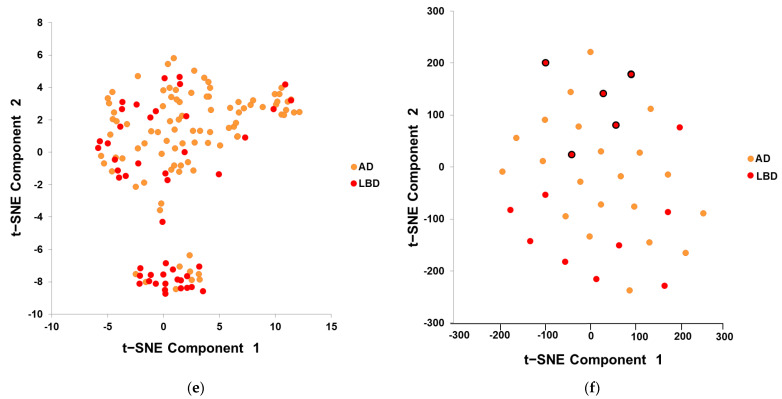
Image features (128 features of FC for each image) visualization of the training and testing data sets after dimension reduction with t-SNE. (**a**,**b**) AD/NC; (**c**,**d**) LBD/NC; (**e**,**f**) AD/LBD.

To distinguish between AD and NC, the features extracted from each Tc-99m-ECD SPECT training image after the deep learning model (“ResNet-50 with ADNI Pretrained + Modified”) are displayed by t-SNE dimensionality reduction, as shown in Figure 3a, and the NC data (blue points) and AD data (orange points) have two clusters, both of which can roughly distinguish AD and NC, which means that the characteristics of AD and NC are distinguishable after training. Figure 3b shows the feature of the images used for testing to distinguish AD and NC, with AD data in the lower left and NC data in the upper right of the figure; however, there was a partial mixing of the clusters. To distinguish between LBD and NC, the feature distributions of the Tc-99m-ECD SPECT image training and testing data sets by the “Original ResNet-50” model are shown in Figure 3c,d. Regardless of the training or testing data set, there was a clear distinguishing ability. To distinguish between AD and LBD, the feature distributions of the Tc-99m-ECD SPECT image training and testing data sets by “ResNet-50 with ADNI Pretrained” model are shown in Figure 3e,f; the trend is similar to Figure 3a,b, but the distinguishing ability is poorer.

### 3.2. Model Testing and Result Evaluation

**Figure 4:**
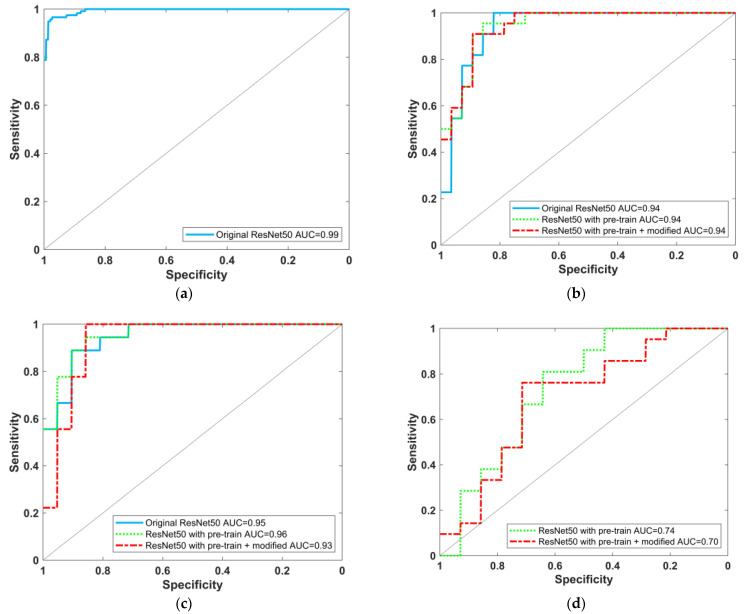
(**a**) ROC curve of ResNet-50 model trained on F-18-FDG PET images for AD/NC; (**b**–**d**) ROC curves of various deep learning models trained on Tc-99m-ECD SPECT images, (**b**) AD/NC; (**c**) LBD/NC; (**d**) AD/LBD task.

The ROC curve of the pretrained model using F-18-FDG PET images is shown in Figure 4a, with an AUC value of 0.99 (95% CI: 0.986–0.997), indicating that the model can be successfully trained to distinguish AD and NC. Furthermore, the ROC curves of the three models for Tc-99m-ECD SPECT images to distinguish AD/NC, LBD/NC, and AD/LBD are shown in Figure 4b–d, with the AUC values and performance evaluation index listed in Table 4, Table 5 and Table 6, respectively. The “ResNet-50 with ADNI Pretrained + Modified” model performed best in distinguishing between AD and NC, with an AUC, sensitivity, specificity, precision, accuracy, and F1 score of 0.94, 91% (20/22), 89% (25/28), 87% (20/23), 90% (45/50), and 89%, respectively. The “Original ResNet-50” model performed best to distinguish between LBD and NC, with an AUC, sensitivity, specificity, precision, accuracy, and F1 score of 0.95, 83% (15/18), 90% (19/21), 88% (15/17), 87% (34/39), and 86%, respectively, whereas the “ResNet-50 with ADNI Pretrained” model was best at distinguishing between AD and LBD, with an AUC, sensitivity, specificity, precision, accuracy, and F1 score of 0.74, 76% (16/21), 64% (9/14), 76% (16/21), 71% (25/35), and 76%, respectively.

## 4. Discussion and Conclusions

Regarding data-driven research, exploring and discovering disease-related features from data has many clinical applications. The prerequisite for a deep learning model to automatically learn about disease features from data is a large amount of data needed to train the model. Radiographic images and retinal optical images have been used in the field of deep learning with excellent results. Moreover, the morphological characteristics of structural images such as CT (computed tomography) and MRI are more similar to the photos in the field of computer vision (CV) than functional images such as PET and SPECT. Therefore, the original deep learning model for CV was first applied to CT and MRI images, with few studies using deep learning techniques for nuclear medicine images to differentiate dementia.

The lack of a large data set of Tc-99m-ECD SPECT images was overcome by using transfer learning technology and reducing model complexity. This study using conventional hardware equipment and about 100 cases of Tc-99m-ECD SPECT image data for each category, reorganized 3D images into three sets of 2D images for data augmentation to improve the accuracy of the training results. The respective prediction of three sets of 2D images from the same subject for ensemble learning improved the accuracy, which is helpful for deep learning training with a small amount of data.

Using t-SNE to display the feature distribution of the data after dimension reduction can intuitively help users understand the pros and cons of data grouping by category after deep learning model training, rapidly identifying the incorrectly predicted cases. For example, Figure 3f shows the feature distribution of the ECD testing data set; there were five LBD data points (red dots with black borders) misjudged as AD. These patients had ages ranging from 61 to 78 years old, had a CDR score of 0.5, and had images of obvious hypoperfusion in suspicious areas. These cases are difficult to evaluate by the model, and consequently, changes in such cases require follow up.

In the overall comparison of the training performance of the AD/NC task, the AUC value was around 0.94, regardless of the training models, with a sensitivity of 91%, specificity of 89%, precision of 87%, accuracy of 90%, and F1 score of 89 for the “ResNet-50 with ADNI Pretrained + Modified” model. These results were better than a previous study [30] which used Tc-99m-ECD SPECT images for a deep learning method to diagnose AD, reporting a sensitivity of 95%, specificity of 75%, and accuracy of 84%. In Table 4, other studies [15,31] used cerebral perfusion SPECT images, but not Tc-99m-ECD, with slightly better results, but their training data consisted of 3D-SSP results and ROI values rather than images. Finally, the use of transfer learning and a modified model resulted in better prediction results, increasing the accuracy by 32%. In Table 5, a comparison of the training performance of the LBD/NC task shows an AUC value higher than 0.93, regardless of training models. It is worth noting that although the training results were quite good (accuracy was greater than 85%), directly using the “Original ResNet-50” model has slightly higher accuracy, implying that using a large number of F-18-FDG PET AD/NC images for pretraining was not very helpful and that LBD features have been retrained with better results. In Table 6, the comparison of the training performance of the AD/LBD task showed that training by the ResNet-50 model failed, and other models had an accuracy of about 70%. The performance of the “ResNet-50 with ADNI Pretrained” model was better than the “ResNet-50 with ADNI Pretrained + Modified” model, but it still seems unable to effectively learn the characteristics of the differences between AD and LBD. As mentioned before [15], the results of 3D-SSP processing have been trained with an accuracy of about 89%, which shows the important role of highlighting the regional differences.

In summary, this study used conventional hardware equipment and a small amount of data to prove the feasibility of successfully training Tc-99m-ECD SPECT images to distinguish between AD and LBD through transfer learning technology and reducing model complexity. However, because AD and LBD have been shown in past studies to often overlap in abnormal areas of the image, as compared with whole-brain image information, cerebral perfusion in certain tissue areas (such as the occipital lobe, cingulate cortex, etc. [14]) can improve the ability to distinguish between AD and LBD. The use of vision transformer (ViT) [32] and attention technology can automatically enhance the learning of more regional details and their relevance, helping to consider whole-brain information while also perceiving the impact of regional changes, and can extend to knowledge-based explainable AI. Furthermore, the experience of the area which concerns doctors can be concatenated into the top layer of the deep learning model to understand the features automatically extracted by the AI model corresponding to the regulation and domain knowledge. Thus, the deep learning model can improve the overall training efficiency of the model and find more important features of differentiating between AD and LBD using a small amount of data. In the future, ViT architecture will be used to improve the effectiveness of training and add physician’s mark information to achieve Knowledge-based Explainable AI.

## Figures and Tables

**Table 1:** Demographic and clinical characteristics of Tc-99m-ECD SPECT data.

Characteristic	NC	AD	LBD
Number of subjects	134	113	61
Age at the time of SPECT (years)	67.0 ± 8.5	74.4 ± 7.0	77.2 ± 5.9
Sex (F:M)	88:46	58:55	25:36
MMSE	27.5 ± 2.4	19.2 ± 5.3	17.6 ± 5.9
CDR	0.22 ± 0.25	0.79 ± 0.39	0.93 ± 0.50

**Table 2:** Demographic and clinical characteristics of F-18-FDG PET data.

Characteristic	NC	AD
Number of subjects	666	667
Age at the time of SPECT (years)	76.4 ± 5.7	76.8 ± 7.5
Sex (F:M)	282:384	268:399
MMSE	28.5 ± 4.0	21.9 ± 5.1
CDR	0.03 ± 0.16	0.83 ± 0.41

**Table 3:** The number and class distribution of all classification tasks in training and testing data sets.

Task	Training Data Set (80%)	Testing Data Set (20%)
ADNI Pretrained AD/NC	549/517 (total: 1066)	118/149 (total: 267)
AD/NC	91/106 (total: 197)	22/28 (total: 50)
LBD/NC	43/113 (total: 156)	18/21 (total: 39)
AD/LBD	92/47 (total: 139)	21/14 (total: 35)

**Table 4:** Comparison of the training performance of ECD data sets in various models for distinguishing between AD and NC.

	Method	Sensitivity (%)	Specificity (%)	Precision (%)	Accuracy (%)	F1 Score (%)	AUC for AD/NC (95% CI)
Proposed (ECD image)	Original ResNet-50 model	90.91 (20/22)	50.00 (14/28)	58.82 (20/34)	68.00 (34/50)	71.43	0.94 (0.82–0.99)
	ResNet-50 model (with ADNI pretrain)	95.45 (21/22)	78.57 (22/28)	77.78 (21/27)	86.00 (43/50)	85.71	0.94 (0.86~0.99)
	ResNet-50 model (with ADNI pretrain + modified)	90.91 (20/22)	89.29 (25/28)	86.96 (20/23)	90.00 (45/50)	88.89	0.94 (0.84–0.98)
Reference (ECD image)	3 layers DNN ^1,+^	95.12	75.00	-	83.51	-	-
	Naive Bayes ^+^	68.29	91.07	-	81.44	-	-
	Decision trees ^+^	78.05	85.71	-	82.47	-	-
	SVM ^+^	82.92	82.14	-	82.47	-	-
Reference (nonECD image)	CNN ^2,^* (I-123-IMP 3D-SSP)	-	-	-	92.39	-	0.94
	ANN ^3,^^ (HMPAO 36 value)	93.80	100.00	-	-	-	0.97

^1^ Deep neural network ^+^ Segovia F et al., 2017 [30]; ^2^ convolutional neural network * Iizuka T et al., 2019 [15]; ^3^ artificial neural network ^^^ Swietlik D et al., 2019 [31].

**Table 5:** Comparison of the training performance of ECD data sets in various models for distinguishing between LBD and NC.

	Method	Sensitivity (%)	Specificity (%)	Precision (%)	Accuracy (%)	F1 Score (%)	AUC for LBD/NC (95% CI)
Proposed (ECD image)	Original ResNet-50 model	83.33 (15/18)	90.48 (19/21)	88.24 (15/17)	87.18 (34/39)	85.71	0.95 (0.83–0.99)
	ResNet-50 model (with ADNI pretrain)	94.44 (17/18)	76.19 (16/21)	77.27 (17/22)	84.62 (33/39)	84.99	0.96 (0.83–0.99)
	ResNet-50 model(with ADNI pretrain + modified)	100.00 (18/18)	71.43 (15/21)	75.00 (18/24)	84.62 (33/39)	85.71	0.93 (0.78–0.99)
Reference (nonECD image)	CNN * (I-123-IMP 3D-SSP)	-	-	-	93.07	-	0.95

* Iizuka T et al., 2019 [15].

**Table 6:** Comparison of the training performance of ECD data sets in various models for distinguishing between AD and LBD.

	Method	Sensitivity (%)	Specificity (%)	Precision (%)	Accuracy (%)	F1 Score (%)	AUC for AD/LBD (95% CI)
Proposed (ECD image)	Original ResNet-50 model	Training unsuccessful					
	ResNet-50 model (with ADNI pretrain)	76.19 (16/21)	64.29 (9/14)	76.19 (16/21)	71.43 (25/35)	76.19	0.74 (0.52–0.90)
	ResNet-50 model(with ADNI pretrain + modified)	76.19 (16/21)	57.14 (8/14)	72.73 (16/22)	68.57 (24/35)	74.42	0.70 (0.47–0.86)
Reference (nonECD image)	CNN * (I-123-IMP 3D-SSP)	-	-	-	89.32	-	0.94

* Iizuka T et al., 2019 [15].

## Data Availability

Data is available from the corresponding authors upon reasonable request.
